# Accuracy of Predicting the Genetic Risk of Disease Using a Genome-Wide Approach

**DOI:** 10.1371/journal.pone.0003395

**Published:** 2008-10-14

**Authors:** Hans D. Daetwyler, Beatriz Villanueva, John A. Woolliams

**Affiliations:** 1 Genetics and Genomics, The Roslin Institute and Royal (Dick) School of Veterinary Studies, The University of Edinburgh, Roslin, Midlothian, United Kingdom; 2 Animal Breeding and Genomics Centre, Wageningen University, Wageningen, The Netherlands; 3 Sustainable Livestock Systems, Scottish Agriculture College, Edinburgh, United Kingdom; Peninsula Medical School, United Kingdom

## Abstract

**Background:**

The prediction of the genetic disease risk of an individual is a powerful public health tool. While predicting risk has been successful in diseases which follow simple Mendelian inheritance, it has proven challenging in complex diseases for which a large number of loci contribute to the genetic variance. The large numbers of single nucleotide polymorphisms now available provide new opportunities for predicting genetic risk of complex diseases with high accuracy.

**Methodology/Principal Findings:**

We have derived simple deterministic formulae to predict the accuracy of predicted genetic risk from population or case control studies using a genome-wide approach and assuming a dichotomous disease phenotype with an underlying continuous liability. We show that the prediction equations are special cases of the more general problem of predicting the accuracy of estimates of genetic values of a continuous phenotype. Our predictive equations are responsive to all parameters that affect accuracy and they are independent of allele frequency and effect distributions. Deterministic prediction errors when tested by simulation were generally small. The common link among the expressions for accuracy is that they are best summarized as the product of the ratio of number of phenotypic records per number of risk loci and the observed heritability.

**Conclusions/Significance:**

This study advances the understanding of the relative power of case control and population studies of disease. The predictions represent an upper bound of accuracy which may be achievable with improved effect estimation methods. The formulae derived will help researchers determine an appropriate sample size to attain a certain accuracy when predicting genetic risk.

## Introduction

Genetic risk of disease is an important component of overall risk of disease in addition to environmental, socio-economic, and behavioral risk factors. Therefore, predicting the genetic risk of disease for an individual is a powerful tool in taking preventative measures against the onset of the disease. Such predictions from genetic testing are relatively straightforward when a disease is caused by one or few genes. However, when a disease is of complex inheritance, the genetic risk of the disease may be associated with many loci, each explaining only a small portion of the genetic variance [1], [2]. In this case, the prediction of genetic risk of disease of a particular individual becomes more challenging. Currently, prediction of risk for complex diseases is based mainly on pedigree analysis but this approach yields predictions of risk that are of low precision; for example predictions would be identical for full siblings without offspring, yet the genetic variation among them accounts for half or more of the genetic variance [3], [4].

The identification of very large numbers of single nucleotide polymorphisms (SNP) has enabled the use of genome-wide association studies (GWA) to detect alleles that are associated with risk for complex diseases [5], such as Type II Diabetes and Crohn's disease [6]. In tandem with this substantive increase of SNP data, several methods for quantifying and/or predicting genetic risk of disease from multiple genes have been put forward [7], [8]. Wray et al.[9] extended these methods by using an GWA approach to estimate the individual genetic risk of disease. Unlike the risk estimates obtained using only pedigree, the estimates resulting from such a GWA approach are more precise by allowing for differentiation among full-siblings. In addition, no pedigree or family history is needed either for estimating risk in one genotyped sample from the population or for predicting risk in a fresh sample. Similar genome-wide methodology has been proposed in animal and plant breeding to estimate additive genetic values for quantitative traits [10], [11]. One critical difference between the two genome-wide approaches is that Wray et al. [9] set a significance threshold for the loci selected for disease prediction, whereas Meuwissen et al. [10] use all loci regardless of whether they affect or not the trait considered. The approach of Meuwissen et al. [10] therefore attempts to achieve the maximum estimate precision of the complete genetic value for a given dataset by including loci that may have too small of an effect to achieve statistical significance, and, thus, reduces the overestimation of allele effects [12].

Wray et al. [9] computed the precision of the individual genetic risk estimates by simulation. While simulation studies are useful in getting initial results on the number of phenotypic records needed to achieve a desired level of accuracy, they are computer intensive and time consuming with large numbers of markers. Most importantly, they do not provide a deep insight on how all variables that affect accuracy interact. Therefore, it is desirable to develop deterministic equations that are responsive to all variables that influence accuracy.

Here we present simple expressions for the genome-wide accuracy of prediction of genetic disease risk. We derive general expressions for continuous traits and the necessary extensions for dichotomous disease traits with data obtained either from population studies or case control studies. The predictions are tested by computer simulation under a variety of parameters influencing accuracy, such as, for example, disease prevalence, heritability and distributions of allele effects and frequencies

## Materials and Methods

### Derivation of Equations

The predicted accuracy that is derived below represents the upper bound that can be achieved when estimating effects in one population sample and then predicting individual genetic risk in another sample from the same population. Throughout this article the accuracy of predicted genetic risk (*r_gĝ_*) is defined as the correlation between true and predicted genetic values. One advantage of using *r_gĝ_* is that the factors influencing it can be clearly derived using the principles of population genetics, as we show below. We will first derive equations that are predictive of *r_gĝ_* for a genome-wide approach with a continuous phenotype, such as height, assuming a population study where individuals are sampled at random. These will then be adapted to predict disease risk for a dichotomous phenotype (‘affected’ or ‘unaffected’) with an underlying continuous liability. The equations are then further adapted to the situation of case control data.

### Continuous phenotype

We will assume that there are *n_G_* potential loci affecting a trait which are independent, biallelic and acting additively, where *n_G_* may be large. These loci may be candidate genes or genetic markers of which a significant proportion may have zero effects. For locus *j*, *j* = 1…*n_G_*, let a randomly chosen reference allele for that locus have frequency *p_j_* and true allelic substitution effect *β_j_*. We shall assume without loss of generality that the distribution of allele frequencies *p_j_* is symmetric about *p* = ½, and likewise that allelic effects *β_j_* are symmetric about *β* = 0. No further distributional assumptions will be made here on *p_j_* and *β_j_*, so for example, many of the allele segregating may have negligible or zero effect. No assumptions are made concerning the covariance between *p_j_* and *β_j_* in the populations sampled. We intend to derive the accuracy of the prediction of the additive genetic value (*r_gĝ_*) of an individual that can be achieved after the measurement of *n_P_* phenotypes.

An estimate of the effect of each allele may be obtained by regression of the phenotypic records on the genotypes one locus at a time because the loci are independently segregating. Assume the population variance of the phenotypes is 1. The estimated allele substitution effect will be 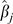 with expectation 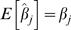, and is obtained by regressing the phenotypes on the observed number of reference alleles in the genotype, denoted *x_ij_* for individual *i* and locus *j* (i.e. *x_ij_* = 0, 1, or 2). The sampling variance of the allele estimate is 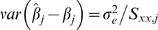 where 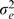 is the residual variance after regression on *x_ij_* and *S_xx_*_,*j*_ = *n_P_var*(*x_ij_*) is the adjusted sums of squares for *x_ij_*. Although not assumed here, when the population is in Hardy-Weinberg equilibrium *S_xx_*_,*j*_ is given by 2*n_P_ p_j_*(1−*p_j_*). For the present, we shall conservatively take 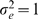, which underestimates the accuracy of the prediction.

Our aim is to predict the accuracy of a new population sample, so we apply the original estimates to a new sample of the same population. Values referring to the second sample will be ‘dashed’, hence individual *i* from the second sample has 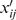 alleles at locus *j*. The additive genetic value of *i* is given by 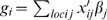 with estimate 
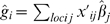
. Then 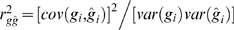. Noting that *ĝ* *_i_* can be re-written as 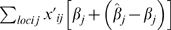 with 
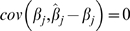
, it is seen that *cov*(*g_i_*, *ĝ* *_i_*) = *var*(*g_i_*) and that 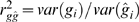. Of these remaining terms, 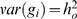, where 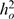 is the observed heritability for the trait, assuming the phenotypic variance is 1. Again using the decomposition 
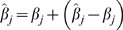
, it can be shown that 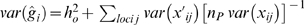, following from (i) the independence of the loci and (ii) the sampling variance of 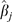 derived earlier. Finally 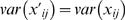, since the second sample comes from the same population, so 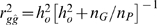, and substituting *λ* = *n_P_*/*n_G_* gives
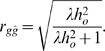
(1)


Therefore accuracy is seen to be a function of the product of the observed heritability 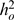 and the ratio of the number of phenotypes recorded to the number of loci involved, *λ*. A second order correction to relax the assumption 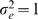 is given in Appendix S1, where it is shown to result in an upward correction to *r_gĝ_* of fractional magnitude 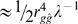.

### Dichotomous disease phenotype

We shall now derive the accuracy of predicting individual genetic risk to disease (*r_gĝ_*) in a random population sample by considering disease prevalence in a liability model [9]. For a disease with prevalence *q*, phenotypes are defined as *s_i_* = 0 for unaffected, and *s_i_* = 1 for affected, so *E*[*s_i_*] = *q* and *var*(*s_i_*) = *q*(1−*q*). Individuals with the highest liability are affected by the disease. Let liability be *y_i_*, scaled so *E*[*y_i_*] = 0 and *var*(*y_i_*) = 1, and *β_j_* is the regression of liability on the number of reference alleles at locus *j*. The linear predictor of *s_i_* on *y_i_* is given by *s_i_* = *q*+*qi_q_y_i_* [13], where *i_q_* equals the mean liability of affected individuals, which we will term the selection intensity [3] corresponding to the prevalence of the disease in the population. Let the slope of the regression of *s_i_* on *x_ij_* be 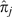, then 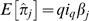, with sampling variance, estimated conservatively using the phenotypic variance *q*(1−*q*)
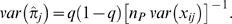
(2)


The coefficients 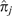 may be rescaled to give estimates 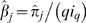, with sampling variance
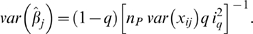
(3)


Repeating the argument outlined above for a continuous phenotype with 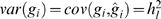, and 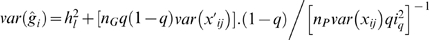, where 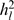 is the heritability on the liability scale. Simplifying terms results in:
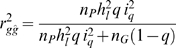
(4)


Robertson and Lerner [14] show that the relationship between additive heritability on the observed scale and the heritability on the liability scale satisfies
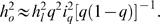
(5)Substitution then results in Equation (1) with 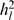 being replaced by 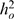:
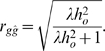
(6)Therefore the dichotomous phenotype study of disease results in an identical formula for *r_gĝ_* as the continuous phenotype provided the heritability used is that for the observed dichotomous scale.

### Case Control Disease Study

The formulae will now be extended to derive the accuracy *r_gĝ_* of a genetic risk prediction when applying a case control design to a dichotomous phenotype. The need for modification of the equations for a case control design comes from the selection of individuals from within the population to achieve a prevalence within the sample of cases and controls of *w*, and where typically *w* = 1/2 with equal numbers of cases and controls. Parameter values post-selection will be ‘starred’. It is assumed in the following without loss of generality that cases are less common than controls in the population so *q*≤*w*≤1/2. Two parameters in particular need to be re-estimated because of the selection practiced: (i) 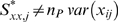; and (ii) the regression of *s_i_* on *x_ij_*, 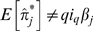. Both these corrections can be made as shown in detail in Appendix S2.

Briefly, assuming no covariance between *p_j_* and *β_j_*, 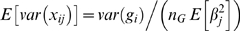. 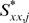 is n_P_var*(*x_ij_*) and so since *n_G_* and 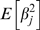 over loci are unaffected by the sampling of cases and controls, *E*[var*(*x_ij_*)] = *E*[*var*(*x_ij_*)]var*(*g_i_*)/*var*(*g_i_*). Appendix S2 shows that using Normal theory 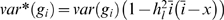. Further 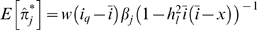, where *x* is the truncation point of a Normal distribution for upper-tail probability *q*, *ī* = *wi_q_*−(1−*w*)*i*_(1−*q*)_.

Approximating 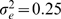 for a binomial trait with probability ½, appropriate for equal numbers of cases and controls, gives 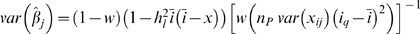, and substituting *λ* results in
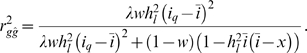
(7)Changing the heritability from the liability scale for a population sample to the observed scale for a population sample using Equation (5) produces
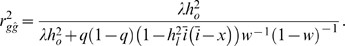
(8)Finally, substituting 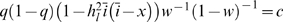, gives
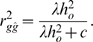
(9)Thus the form of *r_gĝ_* for a case control study shows equivalence to the *r_gĝ_* of continuous and dichotomous phenotypes provided heritability is on the observed scale and the appropriate changes are made in *c* to account for the selection of cases and controls. The value of *c* is 1 in population studies (Equation (6)), where *w* = *q* (and, hence, *ī* = 0). When *q*<*w*<1/2, *c*<1 and there is an increase in *r_gĝ_* compared to a population study with the same *λ*.

### Simulations

Stochastic computer simulations were used to test the deterministic predictions of *r_gĝ_* for a number of parameters affecting the continuous and dichotomous phenotypes. We describe the full simulation method for the continuous trait and then state additional steps that were needed for the dichotomous phenotypes (random population sample and case control). In all scenarios (i) individuals were unrelated; (ii) loci were independent; (iii) all genetic action was additive; (iv) for simplicity, loci were assumed to be in Hardy-Weinberg equilibrium; and (v) each scenario was replicated 100 times, except for case control scenarios with *λ* = 0.02 where 500 replicates were run. Furthermore for initial simulations (vi) allele frequencies were sampled from a uniform distribution corresponding to a common-disease-common-variant hypothesis (CDCV) [15]; and (vii) allele effects were drawn from a reflected exponential distribution which was made symmetric about *x* = 0. Items (vi) and (vii) were modified as described below.

For the continuous phenotypes, the phenotypic variance was 1. True additive genetic values for *n_P_* individuals were calculated as (1−*p_j_*)*β_j_* and −*p_j_β_j_* for the minor and major alleles, respectively, for each of *n_G_* simulated loci, and summing over loci. The value of *n_G_* used in most scenarios was 1000 and *n_P_* varied accordingly, depending on *λ*. Two exceptions were *λ* = 0.02, where *n_G_* = 20,000, and the scenarios in which *λ* was kept constant with *n_G_* = 100. The scale factor of the exponential distribution was chosen to obtain the required additive heritability 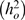. Phenotypic records were simulated by adding independent environmental terms to the true genetic effects drawn from a Normal distribution with mean zero and variance 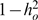. Allele substitution effects 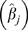 were estimated by regression of *n_P_* phenotypic records on genotypes one locus at a time. A second sample of individuals was then simulated with genotypes based on the same allele frequencies and effects as the original population. The estimated additive genetic values were then computed according to the following model: 
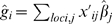
, as described above. Finally, *r_gĝ_* was calculated as the correlation between true and estimated additive genetic values. Bias was also assessed by the slope of the regression of *g_i_* on *ĝ* *_i_*.

The continuous phenotype case was tested for robustness to different distributions of allele frequency and effects, and their correlation. The allele frequencies were also drawn from a beta (U-shape) distribution, consistent with a neutral allele model [16], with parameters alpha = 0.3, and theta = 0.3. Allele effects were also sampled from a normal distribution with mean zero. The effect of having a percentage of loci with zero effects was investigated by setting a proportion of the effects to zero while keeping the overall genetic variance constant. In all cases, the scale factor for the distribution of allele effects was modified to maintain the desired 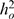.

Further testing of the predictions was done by introducing a correlation between the heterozygosity at a locus and the squared magnitude of the allele substitution effect at a locus. This was done for a uniform distribution of allele frequencies and the reflected exponential distribution of allele effects. This was achieved empirically: if the randomly drawn frequency had heterozygosity greater than the median (i.e. 2*p*(1−*p*)>0.375) then the magnitude of the allele effect was drawn to be less than the median of the distribution of the magnitudes.

The simulation of a random population sample for the dichotomous disease phenotype followed the same structure as above but contained the additional step of treating the underlying continuous phenotype distribution as a liability for the disease with heritability 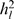 on the liability scale [14]. Therefore, with prevalence *q*, the fraction *q* of the population with the greatest liability were considered to be affected. Therefore allele effects were estimated from the dichotomous phenotype and the accuracy, *r_gĝ_*, was calculated as the correlation between the true and estimated genetic liability for the disease estimated in an independent population sample.

Case control studies were simulated with an equal number of cases and controls (i.e. *w* = 1/2). A dichotomous disease phenotype with sample size *n_P_* was simulated by including an additional selection step which expanded the population size to *n_P_*[2*q_d_*]^−1^. The liabilities were constructed as for the population study of a dichotomous disease, the *n_P_*/2 individuals with the greatest phenotypic liability were considered to be affected cases, and a further *n_P_*/2 were randomly chosen from those remaining as control phenotypes. Allele effects were estimated as for the population studies, and the accuracy was estimated from a randomly-drawn independent population sample of size *n_P_*.

## Results

### Population-wide studies of continuous phenotypes

When allele effects were drawn from an exponential distribution and frequencies were from the uniform, the deterministic formula for *r_gĝ_* was found to predict the simulated data reliably across the wide range of parameters used (Table 1). The prediction errors across all parameters studied were in the range of −1.3 to 4.0% (Table 1).

**Table 1:** Predicted accuracy and percentage prediction error assessed by simulation with disease prevalence = 0.1 (SE range 0.0004–0.0065).

	*h* ^2^ b	*λ* a = 0.02		*λ* = 0.50		*λ* = 1.00		*λ* = 5.00	
		P^c^	%error^d^	P	%error	P	%error	P	%error
C^e^	0.1	0.045	4.0	0.218	3.6	0.301	2.2	0.577	0.4
	0.5	0.100	2.1	0.447	−0.5	0.577	−0.2	0.845	−0.1
	0.9	0.133	−1.3	0.557	0.2	0.688	−0.2	0.905	−0.1
D_P_ f	0.1	0.026	−14.1	0.130	−6.6	0.182	−2.2	0.382	−1.6
	0.5	0.058	−1.1	0.281	0.6	0.382	−1.1	0.679	0.2
	0.9	0.078	−9.8	0.365	1.6	0.485	0.8	0.779	0.2
D_C_ g	0.1	0.043	−0.6	0.209	2.4	0.290	3.5	0.560	−1.9
	0.5	0.089	−4.3	0.407	3.0	0.533	0.8	0.816	−2.9
	0.9	0.112	−20.0	0.490	−0.4	0.622	−0.4	0.872	−3.3

a *λ* = number of phenotypes per number of loci.

b *h* ^2^ = heritability (observed scale for C and D_P_, liability scale for D_C_).

c P = predicted accuracy of estimated additive genetic value.

d % error = percentage prediction error = 100(P−accuracy from simulation)/P.

e C = continuous phenotype.

f D_P_ = dichotomous phenotype, population study.

g D_C_ = dichotomous phenotype, case control study.

The close agreement between the predicted and achieved accuracies is also seen in Table 2 and was maintained when: (i) allele frequencies were drawn from a beta-distribution (% error −0.9 to 0.7); (ii) allele effects were drawn from a normal distribution (% error −0.8 to 5.0); (iii) exponential allele effects were mixed with varying proportions of alleles with no effects, ranging from 0 to 95% (% error 0.1 to 26.6, Table 3); (iv) *λ*'s ranging from 0.02 to 5 were investigated (% error −20.0 to 4.0, Table 1); and (v) the genetic architecture was varied by keeping *λ* constant and changing *n_G_* (*n_G_* = 100, % error 0.1 to 7.6; and *n_G_* = 1000, % error −0.5 to 0.0). It should be noted that the large percentage errors seen when *λ* = 0.02 are due to low *r_gĝ_*, where the absolute difference between the expected and simulated *r_gĝ_* was still less than 0.02. The introduced correlation between heterozygosity and squared substitution effect was tested with *λ* = 1 and *n_G_* = 1000 using the empirical procedure described in the Materials and Methods. With an achieved correlation of −0.36 and an observed 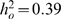, the predicted accuracy from Equation (1) was 0.53, with an error of 1.1% when compared to simulation. In conclusion, it is clear that the deterministic *r_gĝ_* is robust to wide distributional assumptions on the joint distribution of frequency and effect of allele substitution, as predicted from the derivation.

**Table 2:** The effects of different distributions of allele frequency and effects on accuracy in a continuous phenotype with observed heritability = 0.5 (SE range 0.0004–0.0057).

*λ* a	Predicted	Simulated			
		Beta^b^/Nrm^c^	Beta/Exp^d^	Uni^f^/Nrm	Uni/Exp
0.02	0.100	0.095	0.093	0.100	0.097
0.50	0.447	0.442	0.436	0.451	0.450
1.00	0.577	0.577	0.579	0.576	0.578
2.00	0.707	0.709	0.714	0.704	0.709
5.00	0.845	0.849	0.848	0.846	0.846
10.00	0.913	0.914	0.914	0.913	0.912

a *λ* = number of phenotypes per number of loci.

b Beta = beta distribution (alpha = 0.3, theta = 0.3) of allele frequencies.

c Nrm = normal distribution of allele effects.

d Exp = exponential distribution of allele effects.

f Uni = uniform distribution of allele frequencies.

**Table 3:** Accuracy for continuous phenotype when setting 0.95 of *n_G_* a loci to zero (*λ* = 0.02 = 400*n_P_* b/20,000*n_G_*, SE range 0.0042–0.0057).

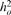 c	0.95 of *n_G_* zero	0.0 of *n_G_* zero	Predicted
0.1	0.057	0.043	0.045
0.5	0.101	0.097	0.100
0.9	0.129	0.135	0.133

a *n_G_* = number of loci.

b *n_P_* = number of phenotypes.

c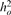 = observed heritability.

**Figure 1:**
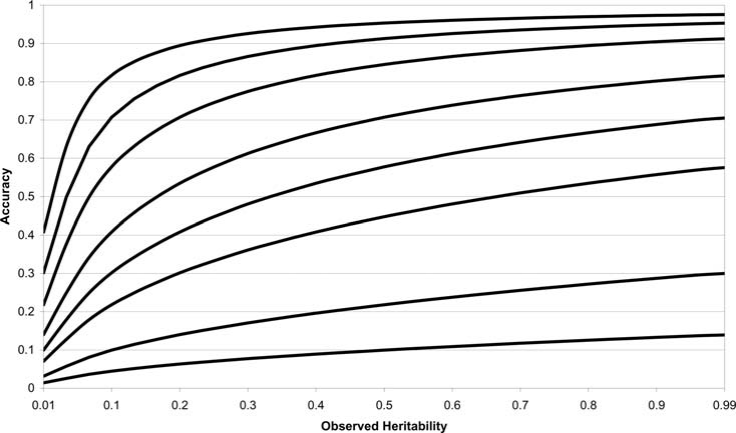
Predicted accuracy of estimated genetic values of a continuous phenotype. Predicted accuracy of estimated additive genetic values of a continuous phenotype as a function of observed heritability and number of phenotypes per genotype tested, *λ* = 0.02, 0.1, 0.5, 1, 2, 5, 10 and 20 from minimum to maximum accuracy respectively.

Therefore the predictions of genome-wide accuracy shown in Figure 1 based on Equation (1) for different values of observed *h* ^2^ and *λ* have wide applicability. For all *λ*, the accuracy was most sensitive to *h* ^2^ when *h* ^2^ was low and this sensitivity was potentiated by higher numbers of phenotypes per genotype tested. The accuracies are functions of *λh* ^2^, so the required *λ* to achieve a given accuracy is proportional to 1/*h* ^2^. Thus, the numbers of phenotypes per genotype need to be twice as high for half the heritability. To obtain accuracies of 0.71, corresponding to predicting half the genetic variance, *λ* = 1/*h* ^2^, and therefore *λ* must be ≥1 because *h* ^2^≤1.

### Population-wide studies on dichotomous disease phenotypes

The form of the predicted accuracy (*r_gĝ_*) is very similar to that for a quantitative trait. Again the prediction of *r_gĝ_* was very good (% error −14.1 to 1.6; see Table 1). The validity of the prediction resulting from Equation (6) was robust to varying disease prevalence over the range of 0.01 to 0.5 (% error −1.9 to 1.4, Table 4). The form of the prediction in Equation (6) is a function of *λ* and the observed additive heritability on a (0,1) scale, but this can be achieved with varied combinations of disease prevalence and underlying heritability of liability. This is shown in Table 5, which also demonstrates that, as predicted from Equation (6), *r_gĝ_* is a function of only 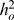 as accuracy remains constant with varied disease prevalence and 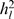.

**Table 4:** Accuracy for a dichotomous disease trait as prevalence varies (a 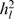, b *λ* = 1, SE range 0.0026–0.0048).

Prevalence	Study Type D_P_ c		Study Type D_C_ d	
	P^e^	% Error^f^	P	% Error
0.01	0.186	−0.8	0.593	−11.1
0.03	0.271	−1.9	0.568	−6.8
0.05	0.317	0.3	0.554	−3.5
0.10	0.382	−0.6	0.533	0.6
0.20	0.444	1.4	0.511	−2.5
0.30	0.473	1.2	0.499	−0.2
0.40	0.487	−0.6	0.493	1.2
0.50	0.491	0.0	0.491	1.4

a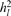 = heritability on liability scale.

b *λ* = number of phenotypes per number of loci.

c D_P_ = population study of dichotomous phenotypes.

d D_C_ = case control study of dichotomous phenotypes.

e P = predicted accuracy of additive genetic values.

f % error = percentage prediction error = 100(P−accuracy from simulation)/P.

**Table 5:** Simulated accuracy of a population study for a dichotomous phenotype as prevalence and 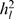 a varies and 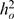 b stays constant (*λ* c = 10, 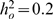, predicted accuracy = 0.816, Equation (4), SE range 0.0025–0.0038).

Prevalence	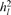	Accuracy
0.05	0.893	0.810
0.10	0.584	0.814
0.20	0.408	0.814
0.30	0.347	0.813
0.40	0.322	0.813
0.50	0.314	0.813

a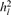 = heritability on liability scale.

b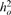 = heritability on observed scale.

c *λ* = number of phenotypes per number of loci.

The predicted *r_gĝ_* of population studies of continuous phenotypes and dichotomous disease phenotypes with an underlying continuous liability follow the same functional form as seen in Equation (6). Therefore, Figure 1 can be used to derive predicted *r_gĝ_* for dichotomous phenotypes as well as continuous phenotypes. However, note that in the liability model, even if liability was fully determined genetically, the additive heritability on the observed scale will never exceed 0.64 (i.e. 4*θ*(0)^2^, where *θ*(*x*) is the standardized normal density function) with the remaining genetic variation appearing non-additive. The corresponding maximum *r_gĝ_* achievable will be reduced and this will be most serious for low *λ*. Even with the most favorable circumstances of *q* = 1/2 and liability 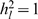, the accuracy will never exceed 0.71 if *λ*<1.56, and it should be expected that *λ* needs to be much greater than this to explain half the genetic variance. This circumstance should not be expected to change when using other disease models than the liability, since the loss of *r_gĝ_* arises from the loss of quantitative information when moving from a continuous genetic value (however defined) to the categorical observation of affected or not.

### Case control studies of dichotomous disease phenotypes

The prediction formula for accuracy of case control studies (*r_gĝ_*) is not a simple function of *λ* and the observed 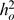, but also depends on both the heritability on the liability scale and the disease prevalence, as seen from Equation (8). Therefore, comparisons require consideration of how *c* in Equation (9) varies. The simulations assumed *w* = 1/2, with equal numbers of cases and controls. Although, as seen in Table 1, the predictions are generally good (% error −20.0 to 3.5), where the large error deviations are again due to low *λ*, there is a trend towards the underestimation of *r_gĝ_* as prevalence becomes low (Table 4).

**Figure 2:**
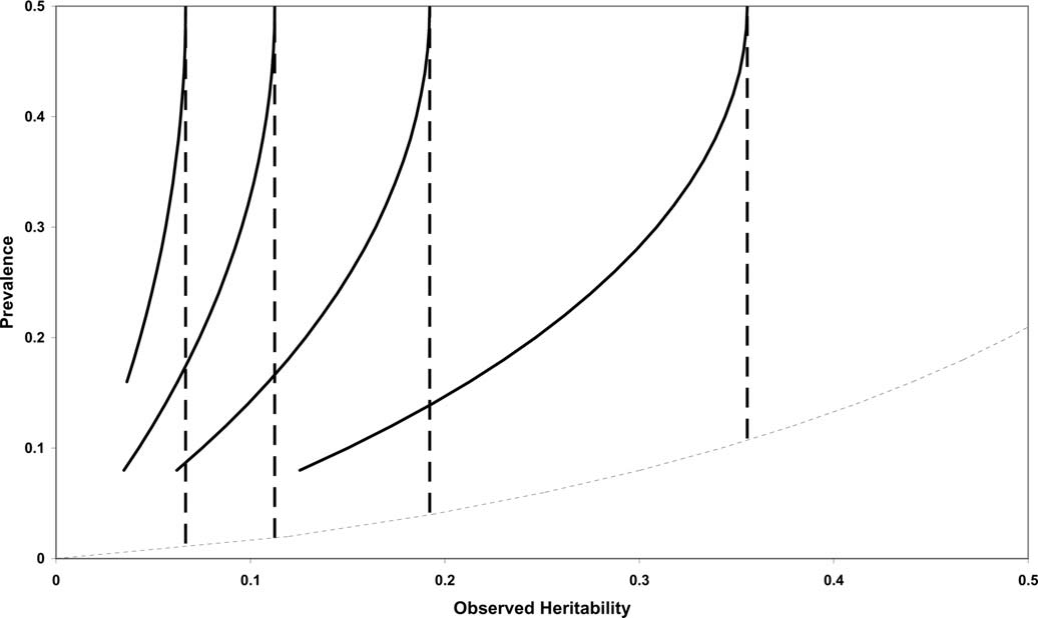
Predicted accuracy of estimated genetic risk from population and case control designs of a dichotomous phenotype. Contour plot of predicted accuracy for varied prevalence and additive heritability on the observed scale, in population studies (dashed vertical line) and case control studies (solid line) of dichotomous phenotypes. Each contour represents a line of constant accuracy, starting from the right 0.9, 0.8, 0.7, and 0.6. The narrowly dashed line is derived from Equation (5) with 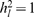, so values below this line are not possible under the liability model.

The value of *r_gĝ_* for case control studies is best illustrated by comparison with population studies of dichotomous disease traits. Figure 2 integrates this information and shows the relationship of prevalence and observed heritability in population and case control studies. Values of *r_gĝ_* below the narrowly dashed line derived from Equation (5) are not possible under the liability model, for example, an observed additive heritability of 0.5 and a prevalence of 0.1 could not exist in the same dataset. Each contour represents an level of constant *r_gĝ_*, where the dashed lines represent a population study and the solid lines denote a case control design with *w* = 1/2. As described above the contours are vertical for population studies as, given 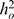, the accuracy is independent of *q*, but for case control studies move towards lower 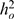 as prevalence decreases. Several clear conclusions on case control studies can be drawn: (i) the overall trend of *r_gĝ_* increasing with more phenotypes per number of genotype holds true for case control studies (Table 1); (ii) population studies and case control studies are equivalent when the prevalence is 0.5 (Figure 2); (iii) a case control study is always more accurate than a population study with the same number of individuals genotyped (Figure 2); (iv) for a constant 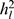, *r_gĝ_* increases as the disease prevalence increases in population studies, since this increases 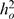, but in case control studies *r_gĝ_* increases as the disease prevalence decreases because of the more intense selection induced by the less prevalent disease (Table 4).

## Discussion

We have derived simple deterministic predictions of *r_gĝ_* in continuous and dichotomous phenotypes using either a population or a case control study and we have shown them to be appropriately responsive to changes in disease prevalence, heritability, and the number of phenotypic records per number of risk loci to be estimated. In addition, the equations have proven robust to changes in allele effect distributions, including different fractions of loci with zero effect and differing allele frequency distributions. Population studies are also robust to covariances between the magnitude of allele effects and heterozygosity, although, in principle, this robustness does not hold for case control studies. This advance in understanding has been used to summarize the influence of critical parameters such as heritability and numbers of phenotypes and risk loci on accuracy of prediction, and also to show the degree to which case control designs can add power to studies.

The approach taken here has been to assume the potential loci affecting the trait are known, and this has an impact that is double edged. First, it allows for a clear quantification of the limitations imposed on *r_gĝ_* by the number of phenotypes obtained, irrespective of marker densities. The information gained by doing so is of equal importance to knowing the number of markers needed for a certain *r_gĝ_* but seems to have received less attention recently. Second, it implies that the predicted *r_gĝ_* are upper bounds for the data obtained, since some loss of *r_gĝ_* will occur through the use of markers which are potentially in imperfect linkage disequilibrium (LD) with loci with effect [17], and the inclusion of candidate loci that may have no effect within the population.

The impact of including these loci with no true effect may be explained by two applications of our formulae. The first application assumed the loci affecting a disease trait are known and thus *r_gĝ_* demonstrates an upper bound on the accuracy; for example, consider *n_G_* = 1000 loci with effects greater than 0, *n_P_* = 10,000 phenotypes and 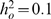, then the predicted accuracy is obtained with *λ* = 10, and will be 0.71. Now consider if those 1000 loci are contained with a set of *n_G_* = 100,000 marker loci, with 99% having zero effect so that now the accuracy is obtained with *λ* = 0.1; our predictive equations remain valid and predict an accuracy of 0.10. From these applications of our formulae it is clear that the approach of estimating loci effects one at a time will inevitably result in low accuracies, and further, adding more marker loci with zero effects while using the same approach will reduce the expected accuracy. The low accuracies predicted accord with the empirical findings from large scale studies of human data that have recently been reported [18]. It is clear that alternative approaches to prediction will be needed to bridge the gap and raise accuracies towards the potential placed by the phenotype collection.

Nevertheless, potential alternative approaches are available and evidence already exists that these approaches may significantly increase predictive accuracy. One approach is to implement model selection approaches. Similarly, improvements in *r_gĝ_* can be achieved by implementing model selection least squares procedures to identify a subset of SNP from which to predict effects [10], [19], or by using more complex procedures to identify a subset to set to zero [20]. Some of these studies [10], [19], [20] also incorporate the use of prior information within Bayesian procedures and demonstrate significant increases in accuracy over least squares. Increasing the number of markers when using priors can increase accuracy because the size of the marker subset chosen stays the same due to the prior but the portion of the genetic variance captured by the markers subset increases [21]. However the use of Bayesian approaches will demand reliable distributions for incorporation into models. Literature estimates informing priors on *n_G_* and the distributions of the effects will become more widely available as GWA studies become more powerful [1], [22]. Full genome-wide methods [10], [11], where genetic risk or additive genetic values are estimated in one step, using all loci simultaneously particularly if they are correlated, might be expected to approach the upper bound of *r_gĝ_* faster than methods which impose significance thresholds and, thus, do not capture all the genetic variation. From the results presented here it may be argued that priors on the numbers of loci positively contributing to the genetic variance will be more critical than those describing the distribution of gene effects.

In this paper we have used a liability model for disease instead of the commonly used log genetic risk model and the impact of doing so is likely to be small for large datasets. For a set of 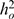 and *q*, an underlying log-risk can be approximated well by a liability [9], [23] and the distribution of effects on the log-risk scale will be transformed to a distribution on the liability scale, and the predictions developed here are not dependent on the distribution of effects. However there is evidence that distinctions may be larger when *q* is very close to zero or one [24].

A critical assumption of the genetic models studied was that the loci acted independently. In humans, most LD stretches for 10 to 30 kb, while some linkage disequilibrium blocks may be >100 kb [25]. The human genome contains 3.1 billion bases [26] and, assuming 2000 known loci contribute to the additive genetic variance, each genomic segment between them would be 1550 kb. This confirms that this model is viable in human. One could apply our formulae by interpreting *n_G_* as the number of independent chromosome segments (i.e. haplotype blocks). The length and, thus, the number of these segments would depend on the amount of LD present in the genome. The number of such segments have been estimated directly from pair-wise LD between markers [27] and closely related measures, such as the number of independent tests on the genome, have been estimated using principle component analysis [28] and have been derived analytically for specific experimental designs [29]. When LD exists, either between markers and risk loci or between risk loci, the predictive efficiency of our equations will be reduced. Modeling the pattern of LD by extension of our formulae would thus be important when many loci are used, as with dense SNP marker maps, or when predicting additive genetic values in other species, such as some livestock populations where the extent of LD is large compared to human [30], [31].

An attraction of molecular predictors of genetic risk compared to pedigree predictors is the potential to apply the predictions more widely within populations and across populations. Obtaining sufficient accuracy within populations can be achieved by the quality and size of sampling, but there are additional factors in play when transfer across populations is being considered. For example, one benefit of genome-wide prediction is that individual allele effects are estimated with a precision that is related to the molecular variation observed at the locus, *var*(*x_ij_*), which determines the contribution of genetic variance when combined with the squared magnitude of effect. This benefit may break down when predictions are transferred across populations. As an illustration, consider a rare allele of large effect which will be relatively imprecisely estimated in the estimation sample, but because the contribution of the locus to total variance is small there is only a small impact upon the accuracy of further predictions within the same population. In a different population, such an allele may have a greater frequency and contribute a greater part of the genetic variance, and, consequently, the predictive accuracy will suffer. Specifically, the ability to transfer predictions will depend on *var*(*x_ij_*) in each of the two populations used for estimation and application, and this in turn depends on both the allele frequency (*p_j_*) and the degree of admixture present in the population. Furthermore, an additional risk of transferability across populations is the presence of epistasis which may differentially influence *β_j_*.

Any directional selection present in the population is likely to introduce a covariance between the magnitude of allelic effect and heterozygosity, since selection promotes the movement of alleles of large effect quickly through intermediate frequencies, where they create large genetic variance, towards extreme frequencies. The predictions of *r_gĝ_* developed make no assumption of the covariance, and hence are robust to such selection in the population prior to estimation in population studies. In contrast, the derivation for the case control study does assume independence of heterozygosity and magnitude (as described in Appendix S2). However, in the limited simulations carried out with such covariances in case control studies, the impact of the breaking this assumption appeared small (results not shown).

Our derivations show that *r_gĝ_* can be reduced to very similar forms for population and case-control studies of continuous and dichotomous phenotypes (c.f. Equations (1), (6) and (9)). The common element affecting *r_gĝ_* for all three equations is the term 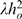, describing the joint effect of *λ*, the number of phenotypic records per locus associated with the trait, and the observed heritability. Increasing either of these improves *r_gĝ_*, but the study shows that the major determinant of the trade-off between these two factors is their product. For a population study 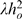 is completely sufficient to determine accuracy, independent of prevalence (*q*) and heritability 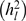 of liability for a dichotomous trait, but for a case control study both *q* and 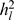 retain some influence on *r_gĝ_* over and above their impact upon 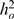. This is because, in a case control study, the term *c* in Equation (9) is adjusting for the selection of the cases and controls, and the strength of selection will depend upon *q*, and its impact on genetic variance will depend on 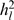.

The predictive equations are a good fit to the simulated values and we have demonstrated, by theory and simulation, that they are independent of allele frequency and effect distributions. The formulae have increased the understanding of the relative differences between predicting *r_gĝ_* in a random sample of a population and in case control studies. The expressions for *r_gĝ_* derived will help researchers design experiments of appropriate size to estimate genetic risk to disease.

## Supporting Information

Appendix S1 (0.56 MB DOC)

Appendix S2 (0.17 MB DOC)
