# Supplementary material for: Accuracy of Predicting the Genetic Risk of Disease Using a Genome-Wide Approach
Source: PLoS One. 2008 Oct 14;3(10):e3395. doi: 10.1371/journal.pone.0003395 (PMC2561058; doi:10.1371/journal.pone.0003395)
Supplement: Appendix S1 — (0.56 MB DOC) [file pone.0003395.s001.doc]

Appendix S1

Equation Error: Reference source not found has been derived assuming . In practice, if , would be derived from a multiple regression and a better approximation would be , where the first term is the environmental variance and in the second the genetic variance is unaccounted for. By replacing in the derivation of Equation (1) with and rearranging the terms using the substitution gives:

This gives a quadratic equation in , and is the solution of which allows for a second order correction of the accuracy. By re-arranging the denominator of Equation so that , and noting the last term may be approximated by , the fractional magnitude of this upward correction to is seen to be . For example if = 10 and = 0.1, then Equation Error: Reference source not found gives and the fractional underestimate is of the order of 0.0125 (i.e. 1.25% error). The same formula can be shown to apply for population studies of dichotomous traits and, analogously, for case control studies.
