# Supplementary material for: Accuracy of Predicting the Genetic Risk of Disease Using a Genome-Wide Approach
Source: PLoS One. 2008 Oct 14;3(10):e3395. doi: 10.1371/journal.pone.0003395 (PMC2561058; doi:10.1371/journal.pone.0003395)
Supplement: Appendix S2 — (0.17 MB DOC) [file pone.0003395.s002.doc]

Appendix S2

Consider the impact on of selection of cases and controls. The selection is equivalent to setting a truncation point of a Normal distribution on the liability scale corresponding to the proportion of affected individuals . This requires sampling the required number of cases with liabilities and the controls with liabilities . In this appendix we will not assume the cases and controls are equally sampled but consider the more general case where are cases and are controls. Prior to setting the truncation point, where is the phenotypic value and . With not small, then we may assume constancy of regression, a property of multivariate normal distributions [31], giving . The can be calculated directly as , where . Therefore and assuming no covariance between and gives the result .

There are three traits to consider, the disease score , liability , and the allele number at locus , *.*  Prior to selection of the cases and controls and the following regression equation holds:

,

with . After selection of cases and controls it is assumed that using a normal approximation the validity of remains, and:

with . Therefore using and gives:

.

Note when , there is no selective sampling and , and , which is identical to in the population study.
